# Optimizing paediatric specialist referrals for short stature in an era of multiple growth hormone indications

**DOI:** 10.1093/pch/pxae025

**Published:** 2024-06-06

**Authors:** Preetha Krishnamoorthy, Nancy Gagné, Rose Girgis, Seth Marks, Zoraida Saoudi, Ian Zenlea, Susan Kirsch

**Affiliations:** Division of Endocrinology and Metabolism, Department of Pediatrics, McGill University Health Center, Montreal, Quebec, Canada; Division of Pediatric Endocrinology, Department of Pediatrics, Centre Hospitalier Universitaire de Sherbrooke, Sherbrooke, Quebec, Canada; Division of Pediatric Endocrinology and Metabolism, Department of Pediatrics, University of Alberta, Edmonton, Alberta, Canada; Section of Pediatric Endocrinology and Metabolism, Department of Pediatrics and Child Health, University of Manitoba, Winnipeg, Manitoba, Canada; Novo Nordisk Canada, Mississauga, Ontario, Canada; Institute for Better Health, Trillium Health Partners, Mississauga, Ontario, Canada; Division of Pediatric Endocrinology, The Hospital for Sick Children, Toronto, Ontario, Canada; Department of Pediatrics, Markham Stouffville Hospital, Markham, Ontario, Canada

**Keywords:** Canadian consensus-based guideline, Referral form, Short stature

## Abstract

The assessment of growth during infancy and childhood is an essential component of paediatric medicine, as atypical growth may point to the existence of an underlying health condition. To reduce morbidity, it is vital that treatment for growth disorders is provided in a timely fashion. However, although there are guidelines regarding referral criteria for short stature in Europe and the USA, there are no such guidelines in Canada. To address this, a series of consultations and workshops with paediatricians, paediatric endocrinologists, family physicians and nurses were held, with the aim of developing a consensus-based set of recommendations for children in Canada showing atypical growth and to identify red flags for children who might benefit from early referral. To achieve this, a referral algorithm and referral form for primary care providers were developed to ensure timely and appropriate referrals, and transmission of the most relevant details to the secondary care consultant.

The monitoring of growth during infancy and childhood is currently standard of care, with normal growth acting as an indicator of optimal health and suboptimal growth as a possible sign of both endocrine and non-endocrine disorders. The early detection of growth disorders is a key objective of clinical practice in order to reduce morbidity, and to offer treatment in a timely fashion (1). Currently, in Europe and the USA, there are guidelines regarding referral criteria for short stature (2,3). However, in Canada there are no formal guidelines for referral criteria or a baseline diagnostic work-up for children with suboptimal growth. This lack of recommended guidelines may result in late diagnoses and a negative impact on a patient’s final adult height. Additionally, most existing referral guidelines focus only on height standard deviation score (SDS). However, this may not be practical as World Health Organization (WHO) growth curves use percentiles in lieu of SDS. Therefore, the use of SDS may lead to unnecessary referrals (when guidelines are applied in a strict manner), or alternatively, may not capture pathology in children whose height SDS is normal (4).

Our goal was to engage paediatricians, paediatric endocrinologists, family physicians and nurses across Canada in a series of consultations and workshops to develop a consensus-based set of recommendations to address the lack of formal guidelines for the referral of children with suboptimal growth in Canada. We also aimed to identify red flags (factors that may prevent morbidity when identified early) for patients with short stature or growth concerns who might benefit from early referral to endocrinology (Figure 1). To achieve this, we aimed to create an easy-to-use tool for primary care providers to ensure timely and appropriate referrals for growth concerns and transmission of the most relevant details to the consultant. Such a tool could potentially allow for better triaging of urgent referrals and timely care.

**Figure 1.**
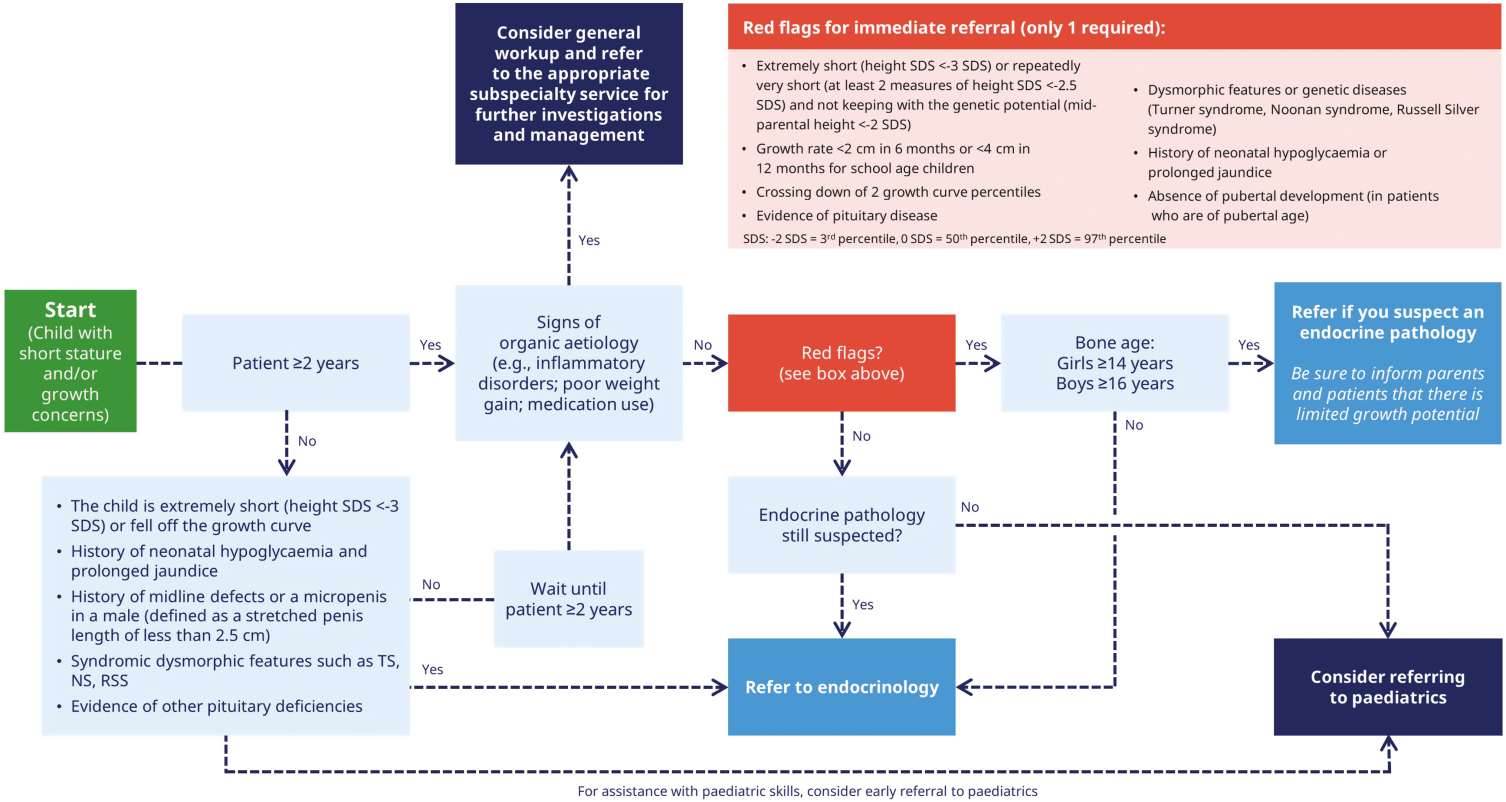
Growth concerns—algorithm for referrals to endocrinology. NS, Noonan syndrome; RSS, Russell-Silver syndrome; SDS, standard deviation score; TS, Turner syndrome

## PROCESS TO DEVELOP REFERRAL RECOMMENDATIONS

This initiative began with a group of 19 participants from across all Canadian provinces except Saskatchewan, Newfoundland and Labrador. The group included paediatric endocrinologists (5), community paediatricians (4), family physicians (2) and paediatric-endocrinology nurses (2), who participated in two workshops. The purpose of the workshops was to generate discussion about best practices for the recognition of short stature in primary care, which included reviewing previously published diagnostic criteria and algorithms from Europe and the USA (6–8). The workshops also aimed to identify necessary clinical information and baseline investigations required for further assessment and referral to specialty endocrinology services. Based on the discussions at these workshops, an algorithm and checklist to support the referral of patients from primary care providers to specialists in an appropriate and timely manner were developed by the consulted physicians according to consensus.

The second phase of the initiative was the consultation phase, during which the proposed tools (algorithm and checklist for referral of short stature) were refined by discussion and consensus, and reviewed in parallel with European and US algorithms (1,4,5,9). In total, 11 individual virtual consultations with paediatric endocrinologists were completed. During the consultations, the need for primary care providers to recognize and refer children with growth concerns was highlighted. This referral may depend on availability. For example, in situations where access to a paediatric endocrinologist is not possible, referral to a general paediatrician is a suitable alternative. Regarding the proposed tools, recommendations would need to be easily accessible and simple to follow, and the referral form would need to be simple and quick to complete. Once these tools were developed, the community paediatricians (4), family physicians (2) and paediatric-endocrinology nurses (2) were provided the opportunity to review the proposals and provide their feedback.

**Figure 2.**
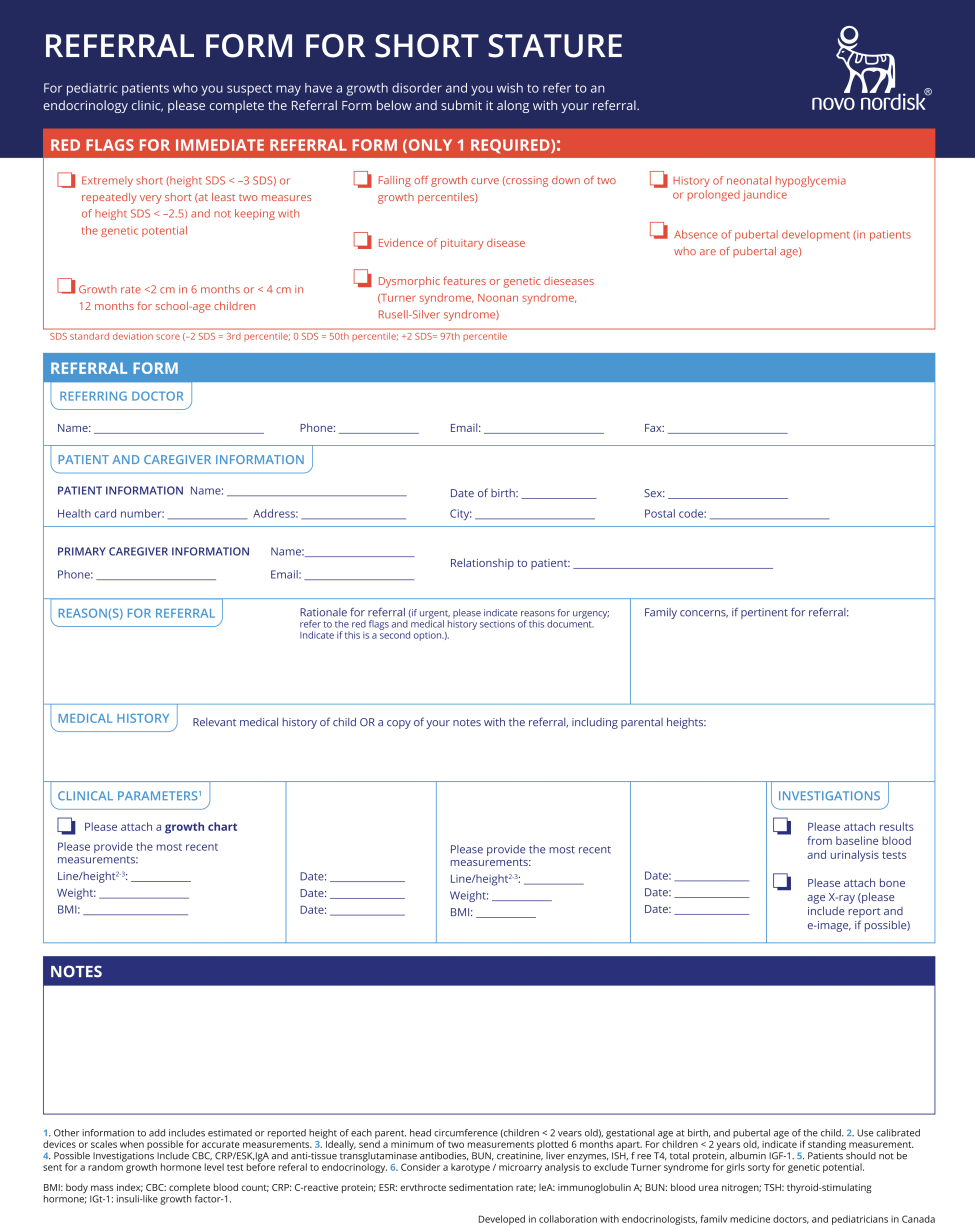
Referral form for short stature

Through this process, a consensus was reached among the consulted physicians regarding an algorithm (Figure 1), to allow for a simplified and improved referral process for short stature in Canada (Figure 2).

## KEY REFERRAL INFORMATION

Key referral information includes a growth curve, parental heights and ‘red flags’ to draw attention to clinical irregularity. The growth curve is the most informative element of the referral, which should be appended to a referral whenever available, and is essential for evaluating growth velocity. Growth velocity was determined to be an important measure in ascertaining whether the patient might have a growth disorder (10). As familial short stature and constitutional delay are the most common normal variants (11), information on the parents’ heights and their age of pubertal development, as well as the patient’s pubertal stage, would also be useful, given that it is important to determine if the child’s growth trajectory is in the height range expected for the child’s biologic family. An estimate of a child’s genetic height potential can be obtained by calculation of the mid-parental height. A discrepancy between the child’s current growth trajectory and the mid-parental height is an indication for further investigation. Paediatrician referral is a valid option at the first signs of growth concerns, because a paediatrician can assist with specialized measurements such as pubertal staging.

### Red flags

Early identifications of ‘red flags’ may prevent morbidity if referral results in subsequent treatment by specialists. For example, in a child with suboptimal growth, hypopituitarism must be considered as it not only impacts growth hormone production but could also result in potentially fatal hypoglycaemia and adrenal insufficiency. Potential clues of congenital hypopituitarism include neonatal hypoglycaemia, prolonged neonatal jaundice, micropenis in a male (defined as a stretched penis length of less than 2.5 cm) or central defects such as septo-optic dysplasia (12). Acquired hypopituitarism may be the result of intracranial pathology. Potential clues of acquired hypopituitarism may include significant headaches, particularly if associated with neurological signs and symptoms, nausea/vomiting or visual disturbance (13). These patients warrant rapid referral to the appropriate specialty (such as emergency medicine, or paediatric neurology/neurosurgery) for further evaluation.

Common syndromes associated with short stature include Turner syndrome, Noonan syndrome and Russell-Silver syndrome. Obvious dysmorphic features or associated anomalies should prompt further genetic testing and early referral (14). Although not all syndromes qualify for growth hormone treatment in Canada, it is still important to make the diagnosis to evaluate for comorbid conditions.

The rapid crossing of major percentiles (e.g., 3rd, 10th, 25th, 50th, 75th, 90th, 97th) on the WHO growth chart for Canada set 2 (15), is possibly indicative of a pathological process, either congenital or acquired. Attention should be made to the percentiles listed on the growth chart being used. For example, if the lowest percentile line is referring to 5th, 3rd, 1st or 0.1st percentile; or if there are few percentile channels, which would take longer to cross than those in charts using more percentile channels. The body mass index growth curve and plotted weight provide additional useful information. For example, deceleration in height percentiles with better preservation of weight percentiles could indicate hypothyroidism or growth hormone deficiency. Weight loss combined with poor linear growth could indicate celiac disease, Crohn’s disease or an eating disorder. Short stature and weight gain could indicate Cushing syndrome.

### Late-age referrals

Although it is essential that diagnoses are completed as early as possible in the life of a patient, patients are frequently referred when their growth velocity has plateaued. For girls who are >2 years old post-menarche or boys who have completed pubertal development, a bone age X-ray should be ordered prior to referral. In girls, at a bone age of 14 years, 98% of growth is complete; in boys, at a bone age of 16 years, 98.2% of growth is complete (16). During this late developmental stage, growth hormone will not be of benefit for increasing final adult height, and patients and families should be made aware that additional height gains are limited at these bone ages (16). A late referral may still be warranted in some circumstances, such as in the absence of a pubertal growth spurt or if the predicted final adult height is significantly below genetic potential (>10 cm below mid-parental height).

### Growth hormone

Many referrals are related to questions about the use of growth hormone. Once referred to endocrinology, patients with suspected short stature will be assessed to determine the aetiology of their condition. Growth hormone has been produced using recombinant DNA technologies since the mid-1980s, and indications for the use of growth hormone in Canada include paediatric growth hormone deficiency (1987), chronic renal insufficiency pre-transplant (1996), adult growth hormone deficiency (1997), Turner syndrome (1997), idiopathic short stature (2006), SHOX haploinsufficiency (2018), Prader-Willi syndrome (2020) and Noonan syndrome (2021). New long-acting, weekly administered analogues of growth hormone have recently been approved in Canada (2022). Long-term safety of growth hormone has been extensively investigated internationally, as well as in Canada (17,18).

## CONCLUSION

Based on expert consensus and consultation with primary care health providers and consultants, we created a referral algorithm and referral form for short stature (Figures 1 and 2, respectively), for use by primary care providers. These tools could potentially facilitate appropriate and timely referrals to paediatric endocrine consultants. We are planning educational webinars to disseminate these tools to primary care providers, paediatricians, and paediatric endocrinologists.
